# Neurocranium thickness mapping in early childhood

**DOI:** 10.1038/s41598-020-73589-w

**Published:** 2020-10-06

**Authors:** Niharika Gajawelli, Sean Deoni, Jie Shi, Marius George Linguraru, Antonio R. Porras, Marvin D. Nelson, Benita Tamrazi, Vidya Rajagopalan, Yalin Wang, Natasha Lepore

**Affiliations:** 1 CIBORG Laboratory, Department of Radiology, Children’s Hospital Los Angeles, Los Angeles, CA USA; 2 Department of Biomedical Engineering, University of Southern California, Los Angeles, CA USA; 3 Voxel Healthcare, LLC, Los Angeles, CA USA; 4 Advanced Baby Imaging Lab, Women & Infants Hospital of RI, Providence, RI USA; 5 Department of Pediatrics, Warren Alpert Medical School at Brown University, Providence, RI USA; 6 Department of Radiology, Warren Alpert Medical School at Brown University, Providence, RI USA; 7 Department of Computer Science, Arizona State University, Tempe, AZ USA; 8 Sheikh Zayed Institute for Pediatric Surgical Innovation, Children’s National Hospital, Washington, DC USA; 9 Departments of Radiology and Pediatrics, George Washington University, Washington, DC USA; 10 Department of Biostatistics and Informatics, Colorado School of Public Health, University of Colorado Anschultz Medical Campus, Aurora, CO USA; 11 Department of Radiology, Keck School of Medicine, University of Southern California, Los Angeles, CA USA

**Keywords:** Paediatric research, Bone development, Biomedical engineering

## Abstract

The neurocranium changes rapidly in early childhood to accommodate the growing brain. Developmental disorders and environmental factors such as sleep position may lead to abnormal neurocranial maturation. Therefore, it is important to understand how this structure develops, in order to provide a baseline for early detection of anomalies. However, its anatomy has not yet been well studied in early childhood due to the lack of available imaging databases. In hospitals, CT is typically used to image the neurocranium when a pathology is suspected, but the presence of ionizing radiation makes it harder to construct databases of healthy subjects. In this study, instead, we use a dataset of MRI data from healthy normal children in the age range of 6 months to 36 months to study the development of the neurocranium. After extracting its outline from the MRI data, we used a conformal geometry-based analysis pipeline to detect local thickness growth throughout this age span. These changes will help us understand cranial bone development with respect to the brain, as well as detect abnormal variations, which will in turn inform better treatment strategies for implicated disorders.

## Introduction

The neurocranium develops rapidly in early childhood. Abnormalities in the shape of the neurocranium during childhood can happen as a result of environmental factors, pathology or injury^1,2^. The neurocranium consists of different plates of ossified bone tissue, which are connected by the soft connective tissue of the sutures and fontanelles^3,4^. This flexible construction permits it to deform and expand to accommodate the rapidly growing brain in early childhood, in addition to allowing the head through the birth canal. The sutures fuse as the child grows, the metopic or frontal suture closing first^5,6^ in the first year of life, while the others including the coronal and sagittal sutures close by age 3. During infancy, between 0 and 2 years of age, the brain surface area grows rapidly and cortical thickness increases. It has been shown that in the first year of life, the brain surface area expands to about 1.80 times of its original area, with the highest expansion in regions including the occipital lobe^7^. Additionally, occipital cortical thickness also increases rapidly between the ages 0 to 9 months^8^. Such growth in the brain drive neurocranium changes. The latter expands rapidly from 25% of its adult size at birth to 90% of its adult size by age 4–5^9,10^, while the brain reaches 95% of its final volume by age 6.

Skull thickness is affected by diseases such as craniosynostosis, where the premature closing of the sutures happens in about 1 in 2000 to 2500 live births^11^, thalassemia, which is caused by abnormal hemoglobin production, Paget’s disease, a disorder causing bone deformities, as well as large arteriovenous malformations^12^. Additionally, deformational plagiocephaly has seen a recent increase in prevalence in early infancy, estimated from 5 to 48%^2^, possibly due to the increase in time babies spend in supine position to prevent sudden infant death syndrome. Studies have shown some association between motor function delays and deformational plagiocephaly^1^. While some non-synostotic deformities may improve with age, positional correction therapy may be needed in other cases. Subsequent brain growth constraints may lead to adverse developmental outcomes if left undiagnosed or untreated within the first years of life^13,14^.

Finally, neurocranial fractures, the leading symptom of injury in infants due to motor accidents, falls and abuse^15^ may also lead to abnormalities. A growth map of the neurocranium may help in addressing correct sutures and bone plates positions in these cases. A previous study has shown that while adult cranial sutures and cranial bones have a similar stiffness, pediatric cranial sutures can deform 30 times more than cranial bone before failing and 243 times more than the adult cranial bone^16^. Due to this, fracture may cause the neurocranium to sustain a major shape change, impacting the brain as well. It is thus imperative for clinicians to accurately diagnose neurocranial deformities early, and to accurately monitor progress and development, both on its own and with respect to the brain. The lack of normative data and quantitative imaging tools for these analyses are major obstacles to the early detection and optimal treatment of conditions of infancy. Additionally, treatment approaches and their success vary widely among institutions and clinicians and are usually based on subjective criteria.

**Figure 1:**
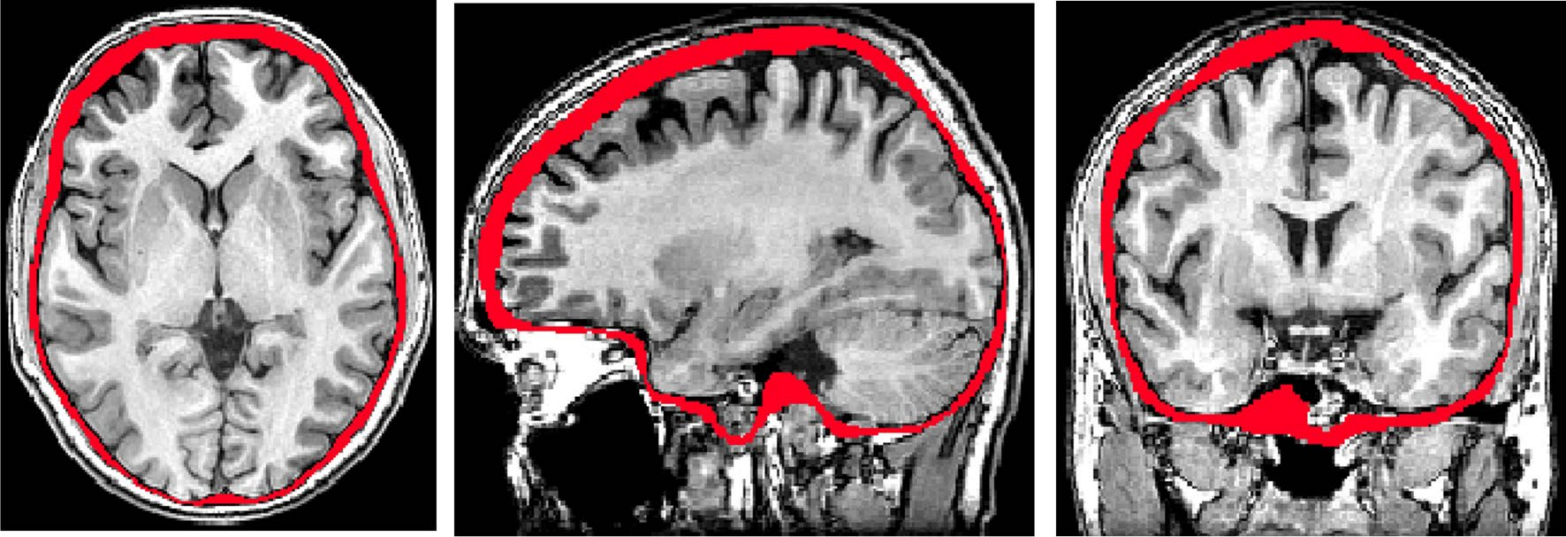
Example of neurocranium segmentation shown on an 18 month old brain.

Computed tomography (CT) images are typically used to diagnose abnormalities of the neurocranium. While the use of this modality is often necessary when diagnosing pediatric patients, studies have also shown a small but significant increase in the risk of long term adverse outcomes such as developing brain cancer or leukemia^17,18,21^, which suggests that alternative imaging procedures without ionizing radiation should be used whenever possible^18^. It is, therefore, inappropriate to subject healthy children to CT scans as a way to collect data for growth maps of shape changes of the neurocranium. Fortunately, this structure can also be delineated on magnetic resonance images (MRI). An example of such a segmentation for an 18-month-old brain is provided in Fig. 1.

MRI has also recently shown value in diagnosis of craniosynostosis through a novel sequence that enhances the bone-soft tissue boundary^19^, as well as to investigate craniosynostosis prenatally^20^.

**Figure 2:**
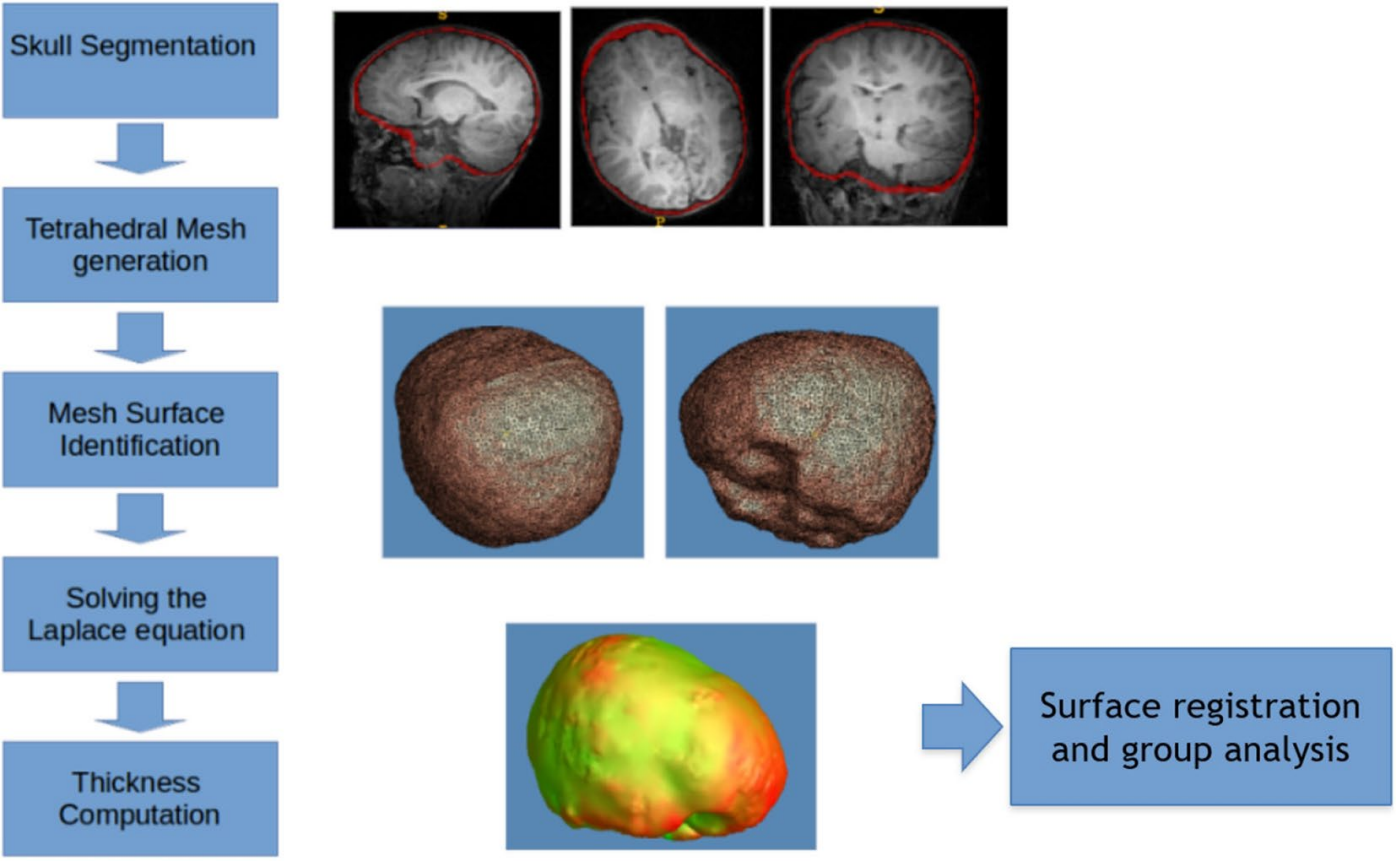
Data analysis flowchart showing the different steps that were involved in the processing.

In the current study, we map normative neurocranial thickness in children between 6 and 36 months of age using a large, preexisting database of MRI scans of healthy children ranging in age from 0 to 3 years old, acquired at the Baby Imaging Lab (https://www.babyimaginglab.com). We focus on thickness because it is an important factor in predicting susceptibility to injury, as well in diseases of the neurocranium such as thalassemia and Paget’s disease. Thickness measurements also have potential for applications in electroencephalogram measurements, that can be erroneous due to variation in neurocranium and scalp thickness^21,22^. In this paper, we use neurocranium masks derived from high resolution MRI brain scans in young children and introduce a new pipeline to investigate neurocranial thickness on MRI data. We first generate tetrahedral meshes, allowing sub-voxel resolution analysis of the thin neurocranium surfaces. We then solve the Laplace equation at each vertex on the neurocranium surface, creating a harmonic field through which we calculate thickness using streamlines. The weighted-SPHARM approach is used to register surfaces, allowing group comparisons. The general flow of processing is shown in Fig. 2^23^. This paper adds to the body of studies involving CT data in ages under 36 months to map neurocranial thickness.

### Method

#### Data

All the methods were performed in accordance with relevant guidelines and regulations. The dataset used includes 56 T1 MP-RAGE MRIs (1.4–1.8 mm^3^) of healthy normal children between the ages of 6–36 months of age at the time of scanning. The number of subjects in each age group is shown in Table 1. The inclusion criteria is as follows: singleton, full term (37–42 weeks at birth) with no abnormalities on fetal ultrasound and no reported history of neurological events or disorders in the infant. Data acquisition details can be found in^24,25^. The study was approved by the Institutional Review Board of Brown University and informed consent was obtained from the guardians of all participants. All data was de-identified before pre-processing and acquired on a Siemens 3 T Tim Trio scanner equipped with a 12 channel head RF array. To minimize intra-scan motion, children were asleep and swaddled with a pediatric MedVac vacuum immobilization bag (CFI Medical Solutions, USA) and foam cushions. Scanner noise was reduced by lessening the peak gradient amplitudes and slew-rates, and using a noise-insulating scanner bore insert (Quiet Barrier HD Composite, UltraBarrier, USA).

**Table 1:** Number of subjects in the different age and gender groups.

Age	Male	Female	Total
6 months	3	2	5
9 months	7	1	8
12 months	3	8	11
18 months	7	3	11
24 months	6	5	11
36 months	8	2	10

#### Pre-processing

**Figure 3:**
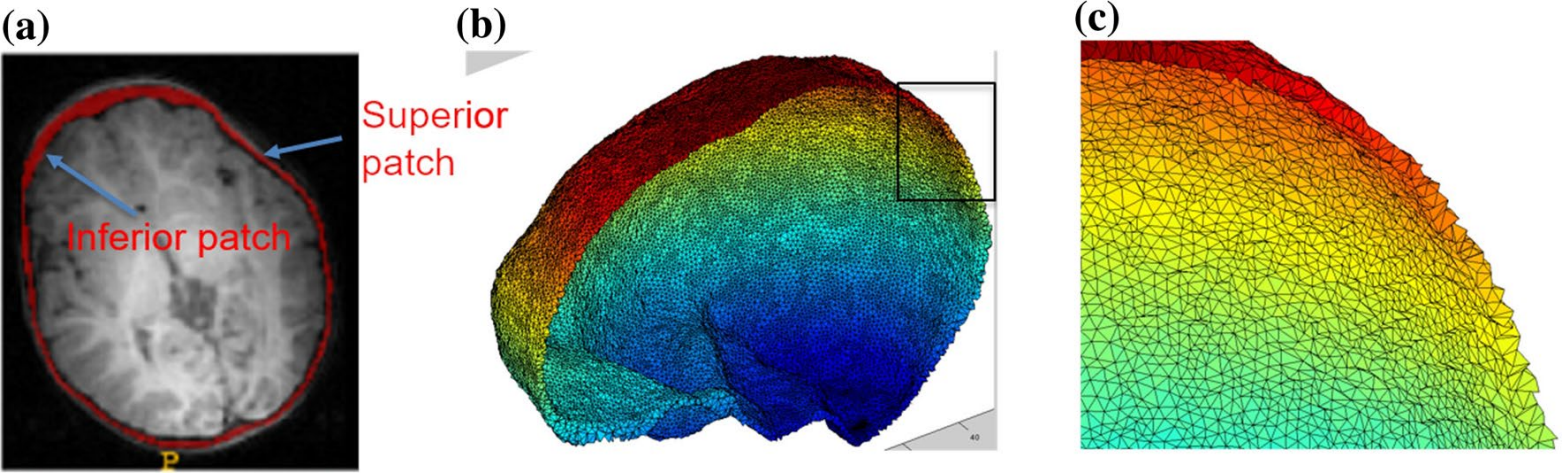
(**a**) Example of neurocranium extracted using FSL. The outer region is the external patch and the inner table is defined as the internal patch. (**b**) Tetrahedral mesh created from the extracted neurocranium using the Iso2mesh toolbox. (**c**) Zoomed in figure of the tetrahedral mesh enclosed in the square. Streamlines are later generated from the outer to the inner patches to compute thickness.

The data was pre-processed as follows. First, the MRI brain volume was skull-stripped using FSL BET^26,27^, and resampled to a 1 × 1 × 1 mm^3^ resolution for consistency throughout processing. Bias correction was performed using the N4 ANTs bias correction tool^28^. The result was then linearly registered to an age-matched custom template, using FSL FLIRT^29,30^ with 6 degrees of freedom. This custom template was previously generated from the same dataset as described by Remer et al.^31^, and also resampled to 1 × 1 × 1 mm^3^ resolution. The transformation that resulted from the registration was saved and then applied to the original dataset with the neurocranium. The resulting registered image (with the neurocranium) was then bias field corrected using the N4 ANTS bias correction tool. Finally, we applied the FSL BET^26,27^ tool to extract the outline of the inner and outer neurocranial surfaces (see example in Fig. 3a). The extracted structure does not include the craniofacial skeleton. Each mask was visually inspected to ensure accuracy, and to eliminate datasets with overlapping voxels between the two surfaces, or those which included areas that were not part of the neurocranium. Note that our very first step involved using skull-stripped images. This was done as we needed to register the data for group comparison. Without removing the neurocranium initially, the high intensity signal from the scalp caused the registration to be imprecise.

#### Thickness calculation

The neurocranium thickness calculation used in this paper involved a similar approach to that used for the corpus callosum in previous studies^32,33^. We used the Iso2mesh toolbox^34^ to create volumetric tetrahedral meshes for the inner and outer neurocranium masks generated by FSL BET^27^. An example is shown in Fig. 3b and c. In short, the thickness is computed from the inner boundary (M0; internal patch) to the vertices on the outer surface mesh (M1; external patch) of a mesh M, by optimizing a string energy function (E):

$$E\left( f \right) = \left\langle {f,f} \right\rangle = \mathop \sum \limits_{{\left[ {vi,vj} \right] \in K}} k(vi,vj)(f\left( {vi} \right) - f\left( {vj} \right))^{2}$$

Here v_i_ and v_j_ are vertices on the tetrahedral mesh, and [v_i_ v_j_] is the edge connecting the two vertices. V and K are the set of all vertices and edges of M, respectively. Each edge [v_i_ v_j_] is assigned a string constant *k*(v_i_ v_j_). Here *k*(v_i_ v_j_) is the discrete harmonic energy^35^ between the two vertices. From our previous work, $k\left(vi,vj\right)=\frac{1}{12} \sum_{ r=1,..,n}lr \mathrm{cot}(\uptheta r)$, where n is the number of tethrahedrons shared by edge [vi,vj], lr the length of edges against edge [vi, vj], and $\uptheta r$ are the associated dihedral angles.

We are looking for the piece-wise linear function $f:V\to {\mathbb{R}}$ that minimizes E. $f$ satisfies Laplace’s equation with Dirichlet boundary conditions. We use the volumetric Laplace–Beltrami operator on the tetrahedral mesh $\Delta$^31^, and fix the values of the function $f$ on the external boundary (B1) as 1, the internal boundary (B0) as 0:

$$\Delta fM\left(vi\right)=0 \forall vi\notin \left(M0\cup M1\right)$$

$$fM\left(vi\right)=0 \forall viM0,$$

$$fM\left(vi\right)=1 \forall viM1$$

If we define *u(s)* is a parametric curve with arc length *s* and *x* is a point on the surface patch we start from, streamlines^36,37^ are constructed to connect the two surfaces by solving the equation below.

$${u}^{^{\prime}}\left(s\right)=\pm \frac{\nabla \mathrm{f}\left(\mathrm{u}\left(\mathrm{s}\right)\right)}{\left|\nabla f\left(u\left(s\right)\right)\right|}, u\left(0\right)=x$$

The thickness is defined as the total arc length of the streamline that traverses the neurocranium from external to internal patches^32,35,36^.

#### Surface registration

Next, we used the weighted-SPHARM approach described in^38,39^ to register each individual subject to a common template. This enabled us to get a surface with the same number of vertices for each subject, allowing us to compare thickness locally between groups. Each neurocranium’s internal and external surfaces were mapped to a sphere using an area preserving and surface flattening algorithm^38,39^. We utilized a template sphere with 40,962 vertices to compute the spherical harmonic components in order to get a SPHARM representation of the surfaces. We utilized non-parametric t-tests with 10,000 random assignments of subjects to groups to compare consecutive age groups. Finally, we conducted a regression analysis to understand neurocranial thickness developmental trajectories. Due to the small number of 6 month old subjects, we did not use it for group comparison. However, this dataset was included in the regression analysis since age in days was used and there was no need to cluster data by age bracket as with the group comparisons.

## Results

**Figure 4:**
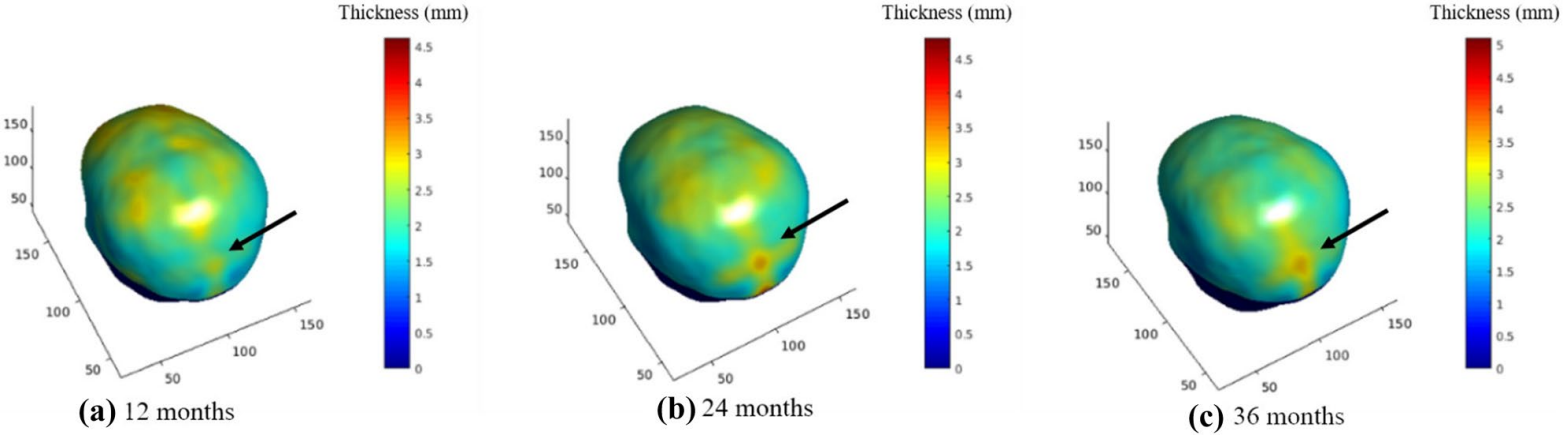
Neurocranial thickness in 3 age groups. (**a**) 12 months, (**b**) 24 months, and (**c**) 36 months. Color bar indicates thickness values. The arrow points to the area of greatest changes, located in the posterior fontanelle region.

Figure 4 shows the neurocranial thickness at a.12 months, b. 24 months and c. 36 months. The color bar goes from dark blue to red, with the latter indicating the thicker regions. Our results show consistent increase in the thickness of the neurocranium, around the midline of the occipital bone under the posterior fontanelle, indicated with the arrows. Internal regions (z-value < 95), including those closer to the air cavities and areas closer or below the cerebellum, were disregarded in this study due to variability in the data, and were set to 0 in the results below.

**Figure 5:**
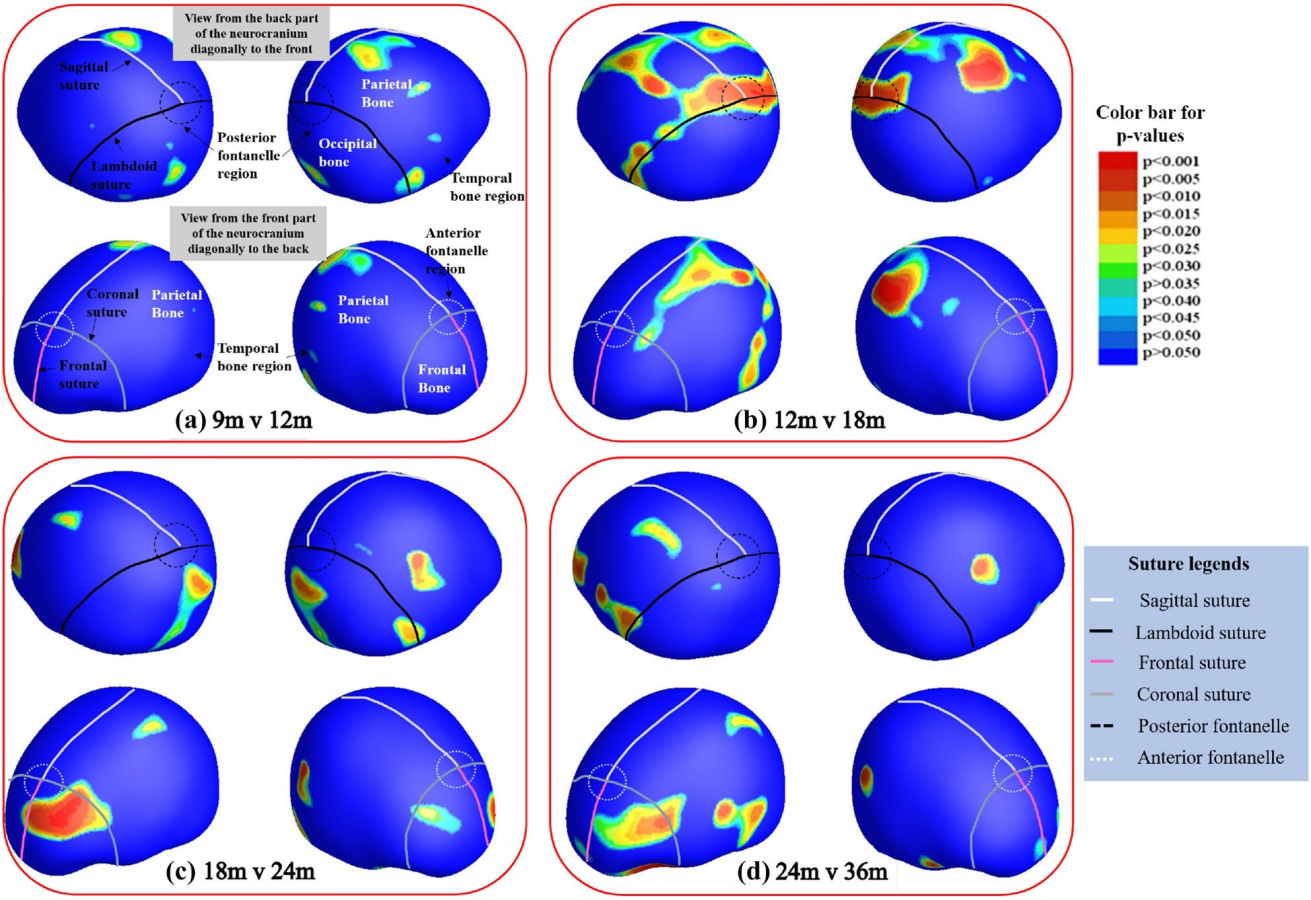
Group differences between each consecutive group. Results were corrected through permutation testing using 10,000 permutations and a significance threshold of 0.05. The color bar indicates the p-values on the image and the sutures are as shown in the legend in the figure: white—sagittal suture, black—lambdoid suture, pink—frontal suture, grey—coronal suture. Anatomical indications are provided only on the (**a**) 9 m v 12 m image for clarity. Additionally, the black circles indicate the posterior fontanelle region and the white ones indicate the anterior fontanelle region. The various bone plates are indicated in white text on the figure. The temporal bone region is below the parietal bone region as indicated by the black arrows in the figure. Each of the 4 figures show locations of significant difference: (**a**) between 9 m and 12 m, (**b**) between 12 m and 18 m, (**c**) between 18 m and 24 m, and (**d**) between 24 m and 36 m.

**Figure 6:**
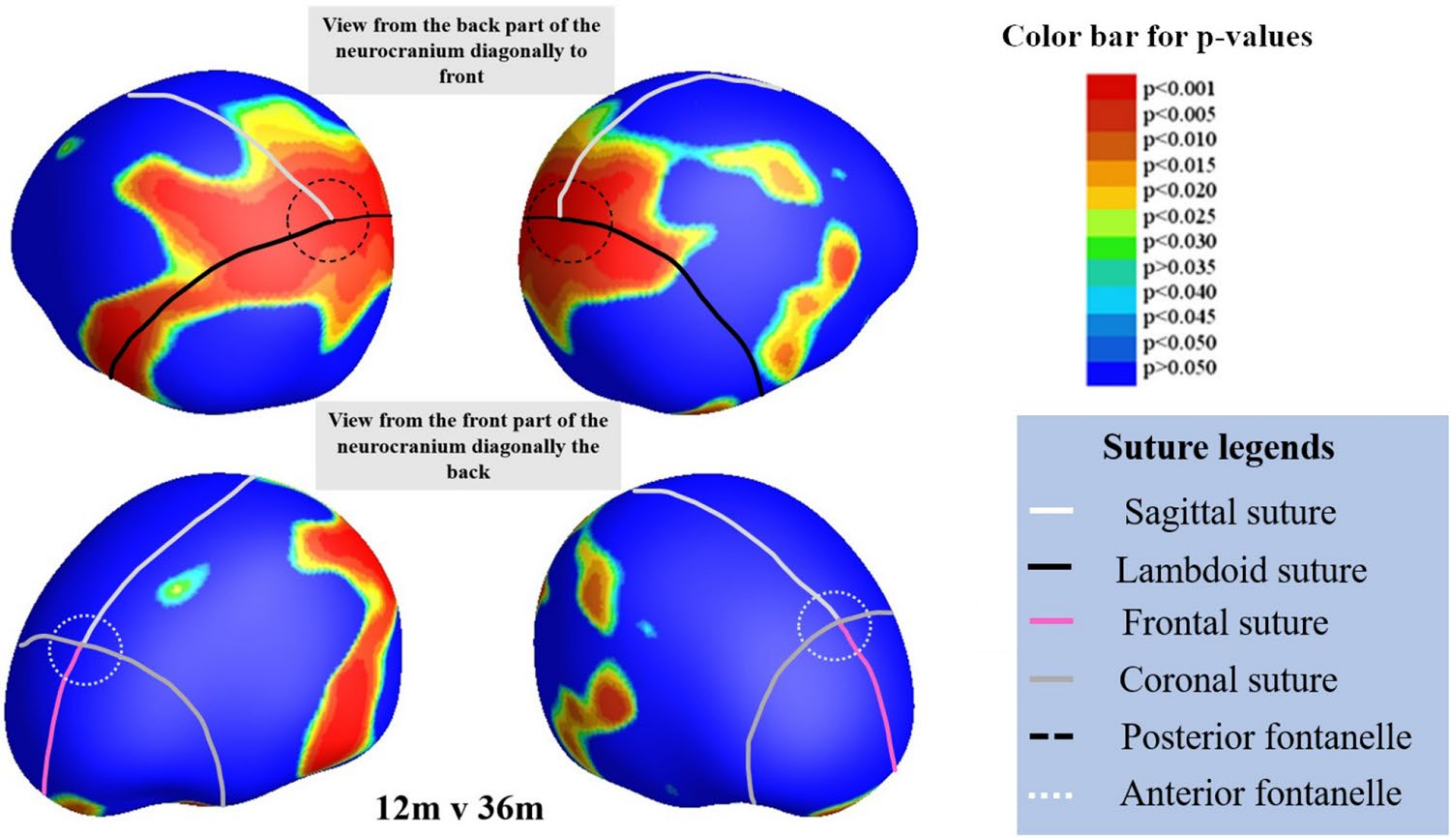
Results of significant difference (p = 0.05) after non-parametric t-tests and multiple comparison correction using permutation testing (10,000 permutations) comparing 12 months and 36 month groups. The largest difference is seen around the lambdoid suture. The color bar indicates the p-values. The lines on the template show the various sutures and fontanelles as described in Fig. 5 above.

Results in Figs. 5 and 6 are of the thickness changes between different age groups. Both figures have indications showing the anatomical landmarks, manually approximated from the MRI template image, such as the various sutures differentiated by color for easier interpretation of the results. Figure 5 represents the p-values showing significant change in thickness of the neurocranium between the ages of a. 9 and 12 months, b. 12 and 18 months, c. 18 and 24 months and d. 24 and 36 months acquired by conducting non-parametric t-tests with 10,000 permutations to a significance value of p = 0.05. The images on top display the posterior part of the neurocranium and those on the bottom the anterior one. Our results show the largest difference between 12 and 18 months for the posterior part of the head, which overlaps with part of the lambdoid suture, where the black line depicts it’s approximate location. Using the same parameters for group comparison, Fig. 6 shows the difference between the neurocranium thickness at 12 months and 36 months. We can clearly see that the differences appear to correspond to the occipital bone under the posterior fontanelle.

**Figure 7:**
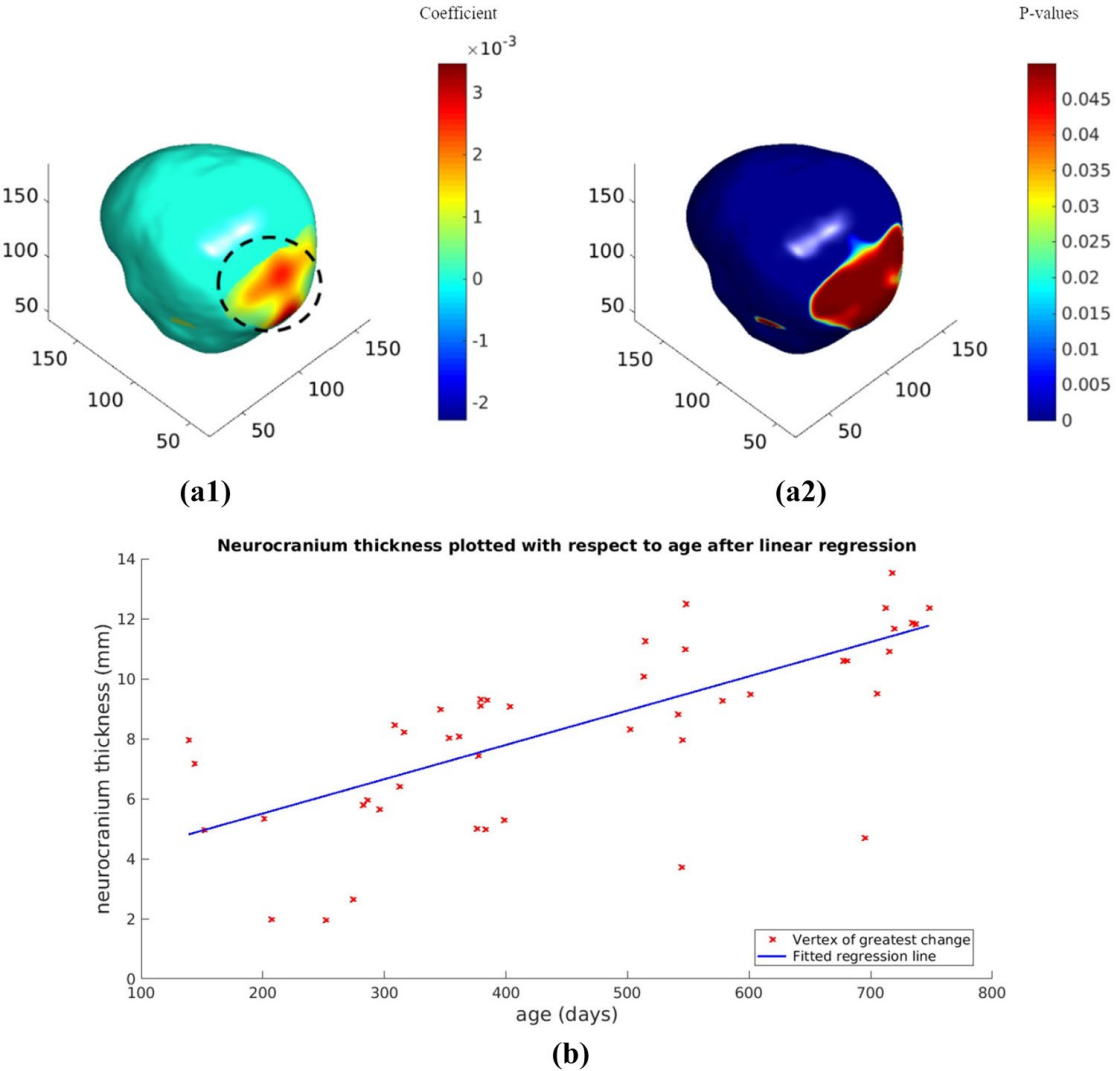
Results of regression showing change over time from 6 to 24 months after FDR correction to significance level of p = 0.05. Red regions indicate the coefficients showing biggest change over time. Regression coefficients shown in (**a1**), and corresponding p-values are shown in (**a2**). Color bar indicates [max (p-value) – p-value] for visualization. Neurocranium thickness change over time for the maximum intensity vertex in the black dotted circle for children between ages of 6 months and 24 months shown in (**b**).

Figure 7 is a linear regression showing the neurocranial thickness trajectory with respect to age starting at 6 months until 36 months. The regions in red show the largest change. We used regression analysis in MATLAB to map the change in neurocranial thickness over time. Figure 7a1 and a2 illustrate the regression coefficients and the p-values of the neurocranium thickness change with respect to the child’s age (in days) from 6 to 36 months. Here, again our results show that the largest change is localized to areas corresponding to the occipital bone of the neurocranium. Using the most significant vertex, we plotted the progression of neurocranium thickness change age in children between the ages of 6 months and 24 months in Fig. 7b. The 36 month data was excluded for this figure as the t-tests showed little difference in neurocranium thickness between the age groups of 24 and 36 months.

## Discussion

Our analysis shows that the biggest change in thickness observed in the neurocranium in early childhood is around the midline of the occipital bone under the posterior fontanelle. Additionally the largest increase in this area is between the ages of 12 m and 18 m and fewer changes are observed thereafter, indicating major development in the posterior part of the head happens in the first 1.5 years of life.

While most studies focus on global measures such as neurocranial volume or head circumference, our study using non-invasive MRI adds to the body of previous studies by providing information on the local thickness changes of the developing neurocranium. Our results give us insight into the development of the neurocranium, as maps shows a clear increase in neurocranial thickness in the posterior part of the head, likely overlapping with the posterior fontanelle.

In a previous study^4^, authors developed a statistical head model for children aged 0–3 from CT images, allowing them to predict neurocranium geometry. Parameters such as age and head circumference were used to predict features such as neurocranium size/shape, suture geometry and neurocranium thickness. The authors showed a greater thickness of the neurocranium in the occipital region in ages 1.5 years and older. In addition, the model also showed that the lambdoid suture closes between the ages of 1 and 1.5 and neurocranium thickness increased in regions corresponding fontanelle after it closes. In Fig. 5b, the most rapid increase in thickness was near the posterior fontanelle and along the lambdoid suture between the ages of 12 and 18 months. Hence, the thickness differences we found in Fig. 5b, agree with this neurocranium growth model^4^. Additionally, we also see a thickness increase in the parietal bones surrounding the sagittal suture between these age groups, consistent with marginal increase in thickness shown in the CT data generated statistical model^4^. Figure 6 shows the thickness difference between 12 and 36 months; the biggest difference around the posterior fontanelle and regions corresponding to the lambdoid sutures. Again, this may be attributed to neurocranium growth after posterior fontanelle fuses.

The largest difference between the 18 and 24 months old cohorts (Fig. 5c) was seen in the anterior part of the head and in the bones surrounding the occipital and temporal lobes. The concentration of points in the temporal region corresponds to a region in between the parietal and temporal bones. Similar differences were also seen when comparing the 24 and 36 month groups (Fig. 5d). Figure 5 shows that the thickness increases non-uniformly, showing larger changes in the posterior part of the neurocranium earlier compared to the anterior region. This non-uniform pattern may also be associated to the posterior to frontal development of the brain itself, as shown in several studies on brain development^40,41^. Additionally, it has also been observed that the occipital gyri, which is in the posterior part of the brain shows slower growth in the 2nd year of life compared to the frontal and parietal lobes^42^.

However, it must be noted that as the differences in Fig. 5c and d are not seen bilaterally, results may also be influenced by the variability of the data or inaccuracies that arise during registration. Alternatively, the patterns may indicate that while most of the neurocranium thickness changes in the posterior and occipital bone area happen prior to 2 years, those of the frontal and temporal bones may continue to evolve even after age 2.

Normal healthy development of the neurocranium and its relationship to brain development is also relevant in understanding deformational plagiocephaly, which occurs due to the continuous placement of an infant in one position the supine position only. Studies have shown that children with deformational plagiocephaly have motor delays in early childhood^1^. Additionally, some children may be predisposed to deformational posterior plagiocephaly due to localized cranial flattening, even though this happens in 13% of healthy newborns. A recent study investigating the association of deformational posterior plagiocephaly and visual field abnormalities showed that in 35% of the 40 infants studied, one or both hemifields were constricted^43^. While these are not causal relationships, and it is possible that deformational plagiocephaly occurs in children who have developmental delays, there are several studies implying that these children face a higher risk of developmental difficulties, language disorders and attention span problems^44,45^. Therefore, it will be beneficial for physicians assessing these children to have a growth model of the normally developing neurocranium to compare to as well as to recognize the relationship between neurocranium and brain development in early childhood to prevent developmental delays from persisting. Additionally, such a growth model may also be beneficial to assess the progress of diseases such as thalassemia, Paget’s disease, to monitor intracranial pressure, as well as use in electroencephalogram measurements^21,22^.

The CT data generated statistical neurocranium models^4^ also show changes in the anterior part of the neurocranium, near the coronal suture, that we were not able to observe in our group comparisons. This may be due to the fact that this change was smaller compared to the posterior part, and we were not able to observe it due to our smaller sample size. This model also shows a small area of the sagittal suture closed after age 2. Thickness may increase gradually after the suture closes and therefore, our dataset may not cover the age range to observe a thickness change that occurs after the suture closes. The data size used in this study and the age range is a limitation as we could reliably extract the neurocranium only from a small number of subjects with the highest quality images, due to the fact that the neurocranium is quite thin in early childhood and difficult to automatically extract from MRI data. To overcome this problem, future studies investigating the neurocranium may benefit from new MRI sequences to enhance the bone-soft tissue boundary^19^.

## Conclusion

In this study, we investigated the growth of the neurocranium across ages 6 to 36 months. Our results showed the biggest change in the posterior part of the neurocranium, where the lambdoid suture lies, between 12 and 18 months, corroborating results of the geometric head model created from CT data^4^. Therefore, it is important in future studies to investigate younger age groups as well as age groups in smaller intervals to build a more detailed timeline of neurocranium growth.

The neurocranium growth model will improve over time by inclusion of more subjects to become a reliable template of neurocranium growth. We show in this pilot study, that using MRI head scans, neurocranium thickness growth can be mapped out to show changes in areas that correspond to previous CT studies. Future work will include a correlation of the neurocranium thickness and parameters acquired from the brain, such as cortical thickness, which will allow us to acquire a more complete picture of the development of the brain and neurocranium. The association between plagiocephaly and neurocranium development, particularly in light in of the fact that our results show consistent group differences in the back of the neurocranium, will also be investigated.
